# Assessing the effects of ocean alkalinity enhancement on marine protozoa: physiological dynamics and transcriptomic responses

**DOI:** 10.1128/aem.00298-26

**Published:** 2026-06-30

**Authors:** Zuyuan Gao, Mengwen Pang, Mingjie Li, Yuzhen Ming, Hongbin Liu, Kedong Yin

**Affiliations:** 1 Southern Marine Science and Engineering Guangdong Laboratory (Zhuhai) 590852 , Zhuhai, China; 2 Department of Ocean Science, the Hong Kong University of Science and Technology, Hong Kong SAR, China; 3 Shenzhen Key Laboratory of Marine Microbiome Engineering, Institute for Advanced Study, Shenzhen University 620539, Shenzhen, China; 4 School of Marine Sciences/ Guangdong Key Laboratory of Marine Resources and Coastal Engineering, Sun Yat-Sen University 26469 , Guangzhou, China; 5 Hong Kong Branch of Southern Marine Science and Engineering Guangdong Laboratory (Guangzhou), Hong Kong SAR, China; University of Nebraska-Lincoln, Lincoln, Nebraska, USA

**Keywords:** ocean alkalinity enhancement (OAE), marine protozoa, heterotrophic nano-flagellates, OAE substances, transcriptome

## Abstract

Ocean alkalinity enhancement (OAE) is proposed as a potential tool to remove atmospheric CO_2_ and mitigate climate change. However, the effects of OAE on marine protozoa remain poorly understood. In this study, we conducted acute and acclimated experiments on two heterotrophic nanoflagellates, i.e., *Cafeteria burkhardae* and *Paraphysomonas longispina*, to investigate their responses to two substances (NaHCO_3_ and NaOH) at low (set ~2,600 µmol L^−1^) and high (set ~4,000 µmol L^−1^) levels, respectively. Our results showed that the two species had similar negative reactions under acute exposure. However, the two species showed different tolerances after acclimation. The growth rates of *C. burkhardae* decreased under all OAE treatments, while its reactive oxygen species accumulated only at the high OAE level. The low-level OAE treatments had no significant effects, but high-level OAE was more adverse for *P. longispina*, which grew more slowly and grazed more, indicating lower growth efficiency. Underlying transcriptomic mechanisms were only analyzed for low-level OAE, which were consistent with the physiological responses. Replication and repair and metabolism-related pathways were significantly inhibited, with translation-related pathways stimulated in *C. burkhardae*. For *P. longispina*, in addition to translation-related pathways, replication and repair, and metabolism pathways were significantly upregulated. Overall, our findings suggest the potential negative effects of OAE on marine protozoa, and the effects can vary depending on the species, level, and substances. Given the critical role of protists in marine ecosystems, these adverse effects raise important concerns about the broader implications of OAE for marine biodiversity and ecosystem stability.

**IMPORTANCE:**

Ocean alkalinity enhancement (OAE) represents a novel approach to mitigate climate change by increasing the ocean’s CO_2_ sequestration capacity. However, the potential ecological and environmental impacts of OAE on marine microorganisms, particularly protozoa, remain poorly understood. This study investigates the responses of two heterotrophic nanoflagellates, *Cafeteria burkhardae* and *Paraphysomonas longispina*, to varying levels of OAE treatments using NaHCO_3_ and NaOH. Our findings reveal significant species-specific differences in tolerance and physiological responses, with implications for microbial community dynamics in marine ecosystems. By employing transcriptomic analysis, we uncover the underlying molecular mechanisms. Ultimately, our study informs the development of sustainable ocean-based climate solutions, emphasizing the importance of considering microbial ecology in environmental management and policy.

## INTRODUCTION

The atmospheric concentration of CO_2_ has been steadily increasing since the Industrial Revolution, which led to global warming and ocean acidification (1, 2). Ocean alkalinity enhancement (OAE), also known as artificial ocean alkalinization, is considered an ocean-based carbon dioxide removal method with some of the most significant potential (3, 4). Generally, there are mainly two ways to increase marine total alkalinity (TA): one is to add alkaline materials directly, and the other is to speed up the slow (timescales of 10k–100k years) natural weathering process (5, 6). Through OAE, large quantities of carbonate or silicate rocks are scattered on land or in the ocean (7, 8). Then, excess proton acceptors over proton donors increase the seawater’s capacity to absorb and store CO_2_ from the atmosphere through chemical sequestration (9). In addition, the addition of alkalinity substances may also lead to pH rising by consuming hydrogen ions and changing the buffering balance (10).

By some *in situ* trial, OAE has been tested by continuous release of 90,000 t of olivine every 3 days over 1 year along the Great Barrier Reef (11), and continuous addition of aqueous NaOH at a coastal site in Tasmania that decreased *p*CO_2_ up to 370 μatm and increased TA by approximately 545 μmol kg^−1^ (12). In addition, some earth system modeling studies have also suggested that OAE could significantly mitigate ocean acidification and climate change (13–15). However, the potential effects of this chemical approach on marine organisms and ecology remain unclear (16, 17). Some pioneering studies have recently noticed this issue and started investigating how OAE influences corals, phytoplankton, and shell-formed organisms. For example, Albright et al. (18) and Feng et al. (19) found that OAE can protect coral reefs from OA threats by reducing the local *p*CO_2_. However, not all organisms and ecosystems can benefit from this due to the use of different OAE substances and the responding organisms. It has been found that limestone-inspired alkalinization had no significant effects on coccolithophore and diatom growth (16). In addition, the build-up of silicic acid and biogenic silica for the diatom community was reduced after alkalinity enhancement (20). Given that, organisms seem to show species-specific responses to OAE, including alkalizing substances and TA levels, but the potential effects of OAE on marine protozoa are still an enigma.

Heterotrophic nanoflagellates (HNFs), an important part of protozoa, are a diverse group of single-celled eukaryotes that show a cosmopolitan distribution in all marine ecosystems (21, 22). As main consumers of bacteria and picophytoplankton, they play critical roles in microbial food webs and marine geochemical cycling by increasing nutrient mineralization (23, 24). HNFs are reported to be sensitive to environmental changes and stress, such as pH, a changeable parameter during OAE. A few studies have indicated the negative effects of elevated *p*CO_2_ on HNF community abundance (25–27), mainly due to the accompanying decrease in pH (28). When seawater pH is increased to 9.5, most protozoa in the natural planktonic community would die after 3 days (29). However, all these studies were based on the community level, where HNF densities were also affected by grazing and competition from other plankton groups. We know little about how specific HNF species respond to OAE and its accompanying consequences.

To comprehensively evaluate the impact of OAE on HNFs, key physiological parameters that serve as indicators of environmental stress were measured, and the underlying transcriptomic mechanisms were elucidated. The specific growth rate is a fundamental metric of fitness and reproductive success, while the ingestion rate reflects feeding behavior and energy acquisition. Gross growth efficiency integrates growth and ingestion processes, revealing the efficiency of converting consumed energy into biomass (30). Notably, the accumulation of reactive oxygen species (ROS) plays a critical role as a direct biomarker of cellular stress (31). ROS, which include species such as superoxide anions, hydrogen peroxide, and hydroxyl radicals, are naturally produced during metabolic processes and serve as important signaling molecules in various cellular pathways (32). However, their overproduction can lead to oxidative stress, particularly when environmental stress disrupts normal metabolic processes such as oxidative phosphorylation and the TCA cycle, while simultaneously impairing the mechanisms responsible for ROS removal (33, 34). This accumulation of ROS can result in damage to proteins, lipids, and DNA, thereby disrupting cellular homeostasis (35). Such oxidative damage can trigger a cascade of cellular responses, including apoptosis and inflammation, further complicating the organism’s ability to cope with environmental stressors (36, 37). Collectively, these physiological metrics offer a multi-level assessment of how OAE-induced chemical changes affect HNFs’ performance, spanning from cellular stress responses to broader ecological implications.

In this study, we selected two ubiquitous HNF species, C*afeteria burkhardae* and *Paraphysomonas longispina*, and conducted factorial experiments with two factors, i.e., TA level and alkaline substrates, in both Acute exposure and Acclimated experiments. We aim to bridge the knowledge gap and address the following questions. (i) How do HNFs regulate their physiological performance in response to acute exposure to OAE? (ii) How will they acclimate to different OAE treatments at physiological and transcriptomic levels? This study demonstrates that OAE can elicit distinct physiological responses in different species of cultured heterotrophic nanoflagellates. Our results provide crucial proof-of-concept that OAE effects are species-specific, which underscores the importance of broadening future research to include a wider range of ecologically relevant taxa to accurately assess the potential impacts of OAE on marine microbial food webs.

## MATERIALS AND METHODS

### Species and culture conditions

**Fig 1:**
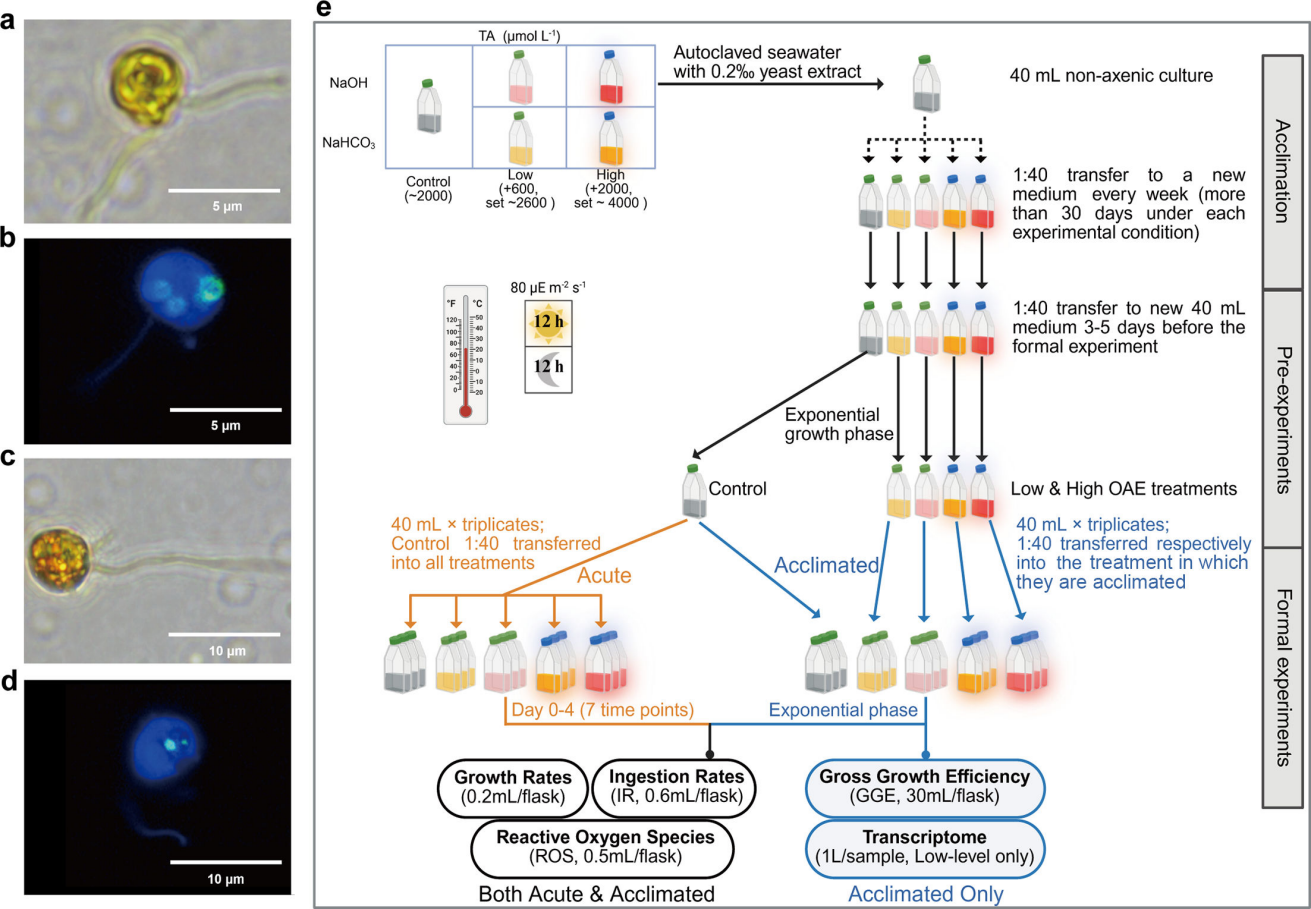
Microscopic images and the experimental design. (**a through d**) Images of Lugol’s iodine solution fixed protozoa species in the bright field under the fluorescence microscope (**a and c**), and DAPI dyed protozoa (blue) with ingested 5-DTAF dyed FLB (green) under a confocal microscope (Leica Stellaris 5, ALEXA 405 and ALECA 488 channel, **b and d**), respectively. *Cafeteria burkhardae* (CB, **a and b**), and *Paraphysomonas longispina* (PL, **c and d**) are shown. Scale bars are applied as references. (**e**) Schematic diagram of the experimental design.

Two heterotrophic nanoflagellate (HNF) species with different body sizes, *Cafeteria burkhardae* (CB, Fig. 1a and b) and *Paraphysomonas longispina* (PL, Fig. 1c and d), were used for experiments. The two species were obtained from the coastal water of Xiamen, China. For daily maintenance, HNFs were cultured in 0.22 μm filtered autoclaved sterile seawater collected from the Southeast Asia Time-series Station (SEATS) in the South China Sea with an average salinity of 32 ± 0.5 and maintained as a non-axenic culture at 23 ± 0.5°C under the light intensity of 80 μmol m^−2^ s^−1^ with a 12:12 light:dark cycle. Autoclaved yeast extract was added to the culturing medium (0.2‰ final concentration) as dissolved organic material for the bacterial prey. The bacterial communities collected by filtration of HNF culture media were transferred 12 h before the HNFs transfer in order to enrich the bacteria to feed HNFs.

### Experimental setup

We conducted both Acute exposure and Acclimated experiments on HNFs, and a 3 × 2 factorial treatment was set up. There were three TA levels: Control (1,955 ± 4.9 μmol L^−1^), Low (600 μmol L^−1^ enhancement, expected 2,600 μmol L^−1^), and High (2,000 μmol L^−1^ enhancement, expected 4,000 μmol L^−1^) with NaHCO_3_ and NaOH. In total, we set five treatments, i.e., Control, low-level NaHCO_3_ addition (Low-C), low-level NaOH addition (Low-OH), high-level NaHCO_3_ addition (High-C), and high-level NaOH addition (High-OH), for each species. The two chemicals were used to configure a 0.2 M stock, and dilution was conducted to achieve each OAE treatment. Before the formal experiment, the two species were maintained in the five treatments of non-axenic cultures semi-continuously by transferring every 5 days and acclimating for more than 30 days (>30 generations).

The main experiments were conducted after the acclimation (Fig. 1e). For the Acute exposure experiment, HNFs kept in a control medium were transferred to the just-prepared culture medium with the five different OAE treatments. In contrast, HNFs acclimated to various treatments were transferred to the corresponding OAE treatment for the Acclimated experiments. All physiological rate measurement samples were cultured in 40 mL culture flasks (EasYFlask, Thermo Fisher). Carbonate chemical samples and RNA samples were cultured in 2 L and 1 L polycarbonate bottles (Nalgene, Thermo Fisher Scientific), respectively. At the beginning of each experiment, the initial densities of CB and PL were controlled around 2 × 10^4^ mL^−1^ and 5 × 10^3^ mL^−1^, respectively (Table S1).

### Total alkalinity and pH measurement

TA and pH changes with time were considered similarly for the Acute and Acclimated experiments. Samples of TA and pH were collected after the 30-day acclimation. Every day, 50 mL of samples was taken from each bottle and filtered by GF/F filter (0.7 μm pore size, Cytiva, Whatman) to remove all the nanoflagellates and most bacteria. All samples were measured by an ALKALINITY TITRATOR (AS-ALK2, APOLLO SciTech) paired with an ORION STAR A211 pH Meter (Thermo SCIENTIFIC) as soon as we finished the filtration. Other carbonate chemistry parameters, including Dissolved Inorganic Carbon (DIC), *p*CO_2_, HCO_3_− concentration, and CO_3_^2-^ concentration, were calculated using the Seacarb package (38) in R.

### Growth-related measurement

A 1 mL protozoa culture was sampled at seven time points for the Acute experiment and every day for the Acclimated experiment during the whole incubation period. Samples were fixed in each experimental triplicate with acidic Lugol’s iodine solution (5% final concentration) and enumerated by an ECUPSE Ti2-U fluorescence microscope (NIKON, Japan). The specific growth rate (μ, d^−1^) was calculated as follows:


(1) $$\begin{array}{c} \mu =\frac{\ln\left(N_{2}\right)-\ln\left(N_{1}\right)}{t_{2}-t_{1}}\; \end{array}$$

in which *N*_1_ and *N*_2_ (cell mL^−1^) are the cell abundance at the first sampling time *t*_1_ and the second sampling time *t*_2_, respectively, and hence, *t*_2_ – *t*_1_ (d) was the sampling interval. For the Acclimated experiment, the highest *μ* among all the culturing days was chosen as *μ*_max_.

### Ingestion rate estimates

The grazing experiments were carried out using fluorescently labeled bacteria (FLB; *Vibrio* sp.) as prey (39) in the 40 mL cultures. Each time, as soon as we collected the HNF cell samples, we added FLB into all cultures with a final density of around 1 × 10^5^ FLB mL^−1^ and incubated them under dark conditions to avoid fluorescence extinguishing. After 1 h of incubation, 0.6 mL of the samples was taken from every flask and fixed with Paraformaldehyde solution (Sigma-Aldrich, Germany) for 30 min. For the total bacteria density, 0.2 mL of subsamples was dyed with SYBR Green I (final concentration 1×; Sigma-Aldrich, Germany) for 1 h in the 37°C water bath (40). All the bacterial cells were counted by a CytoFLEX LX flow cytometer (Beckman Coulter, Inc., USA) with negative controls. The corresponding predator abundances were the same as in the growth experiments. The ingestion rates (IR, bacteria protozoa^−1^ h^−1^) were estimated by calculating the number of FLB existing in our culture medium based on Tsai et al. (41):


(2) $$\begin{array}{c} IR_{=}\frac{N_{flb0}-N_{flbt}}{N_{pro}}\times \frac{1}{t}\times \frac{N_{tb}}{N_{flb0}} \end{array}$$

where *N*_flb0_ and *N*_flbt_ (FLB mL^−1^) are the density of FLB in the culture medium at the beginning and end, respectively. *N*_pro_ (protozoa mL^−1^) is the cell density of the protozoa predators. *N*_tb_ (bacteria mL^−1^) represents the abundance of total bacteria during the experiment. *t* (h) was the duration.

### Gross growth efficiency estimates

Since HNFs acclimated with different OAE treatments were believed to have a stable status, gross growth efficiency (GGE) estimation was only conducted for the Acclimated experiment. Acclimated HNFs and bacteria were cultured in 40 mL flasks, and 30 mL was collected during the exponential phase. The calculation was based on bacterial biovolume consumed relative to the volume of protozoa produced (42). To calculate protozoa carbon biomass, the CB cultures were filtered onto precombusted GF/C filter (1.2 μm pore size, Cytiva, Whatman), and the PL cultures were filtered onto precombusted GF/D filter (2.7 μm pore size, Cytiva, Whatman) with filtrates collected. Then, the filtrates were filtered by precombusted GF-75 filter (0.3 μm pore size, Sterlitech Corporation, WA, USA) for bacteria carbon biomass calculation. Particulate organic carbon on the membrane filters was analyzed by an Elemental analyzer (vario EL CUBE, Germany) with the use of CN mode. The GGE (%) was calculated as follows:


(3) $$\begin{array}{c} GGE=\frac{G}{I}\times 100\%=\frac{POC_{pro}\times \mu_{max}}{POC_{bac}\times IR_{max}\times 24}\times 100\% \end{array}$$

where *G* (pg C protozoa day^−1^) and *I* (pg C protozoa^−1^ h^−1^) were the organic carbon used for growth and acquired from ingestion, respectively. POC_pro_ (pg C protozoa^−1^) and POC_bac_ (pg C bacteria^−1^) were, respectively, the particulate organic carbon of each protozoan and bacteria cell. *μ*_max_ (day^−1^, equation 1) was the maximum growth rate for each protozoa species in each OAE treatment we calculated from the growth experiment, and IR_max_ (bac protozoa^−1^ h^−1^, equation 2) was the ingestion rate related to *μ*_max_.

### Reactive oxygen species measurements

To investigate the intracellular oxidative stress, reactive oxygen species (ROS) were measured by adding 2′,7′-dichlorofluorescin diacetate (H2DCFDA) probe (Reactive Oxygen Species Assay Kit, Biosharp, China) with a final concentration 1μM according to the manual. After the collection of HNF cells every day, 0.5 mL subsamples were taken out and strained with the probe for 1 h at room temperature in the dark. The fluorescence value was measured using a CytoFLEX LX flow cytometer (Beckman Coulter, Inc., USA) at the FITC channel. Cultures without dye were used as the negative controls, and cultures added Rosup (final concentration 0.5 g/mL) and dyed with H2DCFDA were set as the positive controls. The fluorescence value was normalized by the value of the control at each sampling time point.

### RNA extraction, library construction, and sequencing

The transcriptomic analysis was conducted for the low-level and control treatments because the low-level enhancement is more realistic and practical in the ocean (6, 19). After the acclimation, 1 L culture samples (over 2 × 10^5^ cells mL^−1^) of each replicate were filtered onto a polycarbonate membrane with 2 μm pore size (Cytiva, Whatman) and frozen in liquid nitrogen. Total RNA was extracted using PureLink RNA Mini Kit (Thermo Fisher Scientific, MA, USA) according to the protocol. RNA integrity was assessed using the RNA Nano 6000 Assay Kit of the Bioanalyzer 2100 system (Agilent Technologies, CA, USA). Total RNA was used as input material for the RNA sample preparations. Messenger RNA was isolated by utilizing poly-T oligo-linked magnetic beads. Fragmentation was carried out using divalent cations under elevated temperature in First Strand Synthesis Reaction Buffer (5×). First-strand cDNA was synthesized using random hexamer primer and M-MuLV Reverse Transcriptase (RNase H-). Second Strand cDNA synthesis was subsequently performed using DNA Polymerase I and RNaseH. Remaining overhangs were converted into blunt ends via exonuclease/polymerase activities. After adenylation of the 3′ ends of DNA fragments, Adaptors with hairpin loop structures were ligated to prepare for hybridization. The purification of cDNA fragments was conducted with AMPure XP system. Then, PCR was performed with Phusion High-Fidelity DNA polymerase, Universal PCR primers, and Index (X) primer. At last, PCR products were purified (AMPure XP system), while library quality was assessed using an Agilent Bioanalyzer 2100 system (Agilent Technologies, CA, USA). The quantified libraries were combined and sequenced using a NovaSeq 6000 system (Illumina, CA, USA), and 150-bp paired-end reads were generated. For each sample, 15 Gb of raw data was obtained for the RNA sequencing.

The analysis of the RNA sequencing followed Chen (43). Raw reads from RNA-seq triplicates were trimmed using Trimmomatic v0.39 (44). In this step, clean data were obtained by removing reads containing adapters, reads containing poly-T, and low-quality reads from the raw data using the “LEADING:3 TRAILING:3 SLIDINGWINDOW:4:15 MINLEN:36 TOPHRED33” parameter. Then, the clean reads were *de novo* co-assembled by Trinity v2.14.0 (45). Similar transcripts were grouped using CD-HIT (46) with the “-c 0.95” parameter, resulting in a single representative transcript being selected for each cluster. The quality of transcriptomic assembly was estimated using BUSCO v5.4.4 (47) with the lineage data set “stramenopiles_odb10.” TransDecoder v5.5.0 (48) was used to detect the longest open reading frame for each transcript. Then, the transcript was annotated against the Kyoto Encyclopedia of Genes and Genomes (KEGG; 49) using Diamond v2.0.4 (50) with “-sensitive -e 1e-20” parameters. The trimmed transcripts were then aligned to the transcriptomic assembly using Bowtie2 v2.4.4 (51). The read counts of all genes were summarized by featureCounts v2.0.0 (52) based on the results (SAM files) from Bowtie2 with “-M, -O, -fraction” parameters.

### Statistics

All statistical analyses were performed in R 4.3.2 (53), and the significance level was 0.05.

For acute exposure experiments, mixed-effects models were used using “lmer” function, and the differences among treatments during the exposure time period were detected with “emmeans” function.

For acclimated physiological rates analysis, Shapiro-Wilk normality test was conducted using the “Shapiro.test” function. For the normally distributed parameters, one-way ANOVA was implemented, with Levene’s test passed (*P* > 0.05). The Tukey’s Honest Significant Difference test (Tukey HSD test) was then conducted to examine differences among treatments. The “multcompView” function was used to provide different alphabets for significant differences. For the physiological rates with abnormal distribution, we used Kruskal-Wallis test by the “Kruskal.test” function to evaluate the differences. Dunn-Bonferroni test was then conducted to show differences among treatments, and the “cldList” function was used to provide alphabets.

For the transcriptomic analysis, Principal Coordinates Analysis was implemented using the “vegdist” function with “bray” as the method. To identify differentially expressed genes (DEGs), we used the *edgeR* v.3.30.3 R package (54). Only genes with an adjusted *P*-value < 0.01 and |log2 (foldchange)| > 1 were regarded as DEGs. KEGG enrichment analysis was conducted using the “enricher” function in the clusterProfiler v.2.16.1 R package (55). PERMANOVA was implemented with the “adonis” function.

## RESULTS

This study examined the physiological and transcriptomic responses of two heterotrophic nanoflagellate species to simulate ocean alkalinity enhancement using sodium bicarbonate and sodium hydroxide at two concentration levels. The experimental design assessed both acute exposure and longer-term acclimation over 30 generations, measuring key responses in growth, ingestion, metabolism, and gene expression. The following results detail the chemical dynamics during exposure and the species-specific, substance- and concentration-dependent impacts on HNF physiology and cellular processes.

### TA and pH during the acclimated experiment

**Fig 2:**
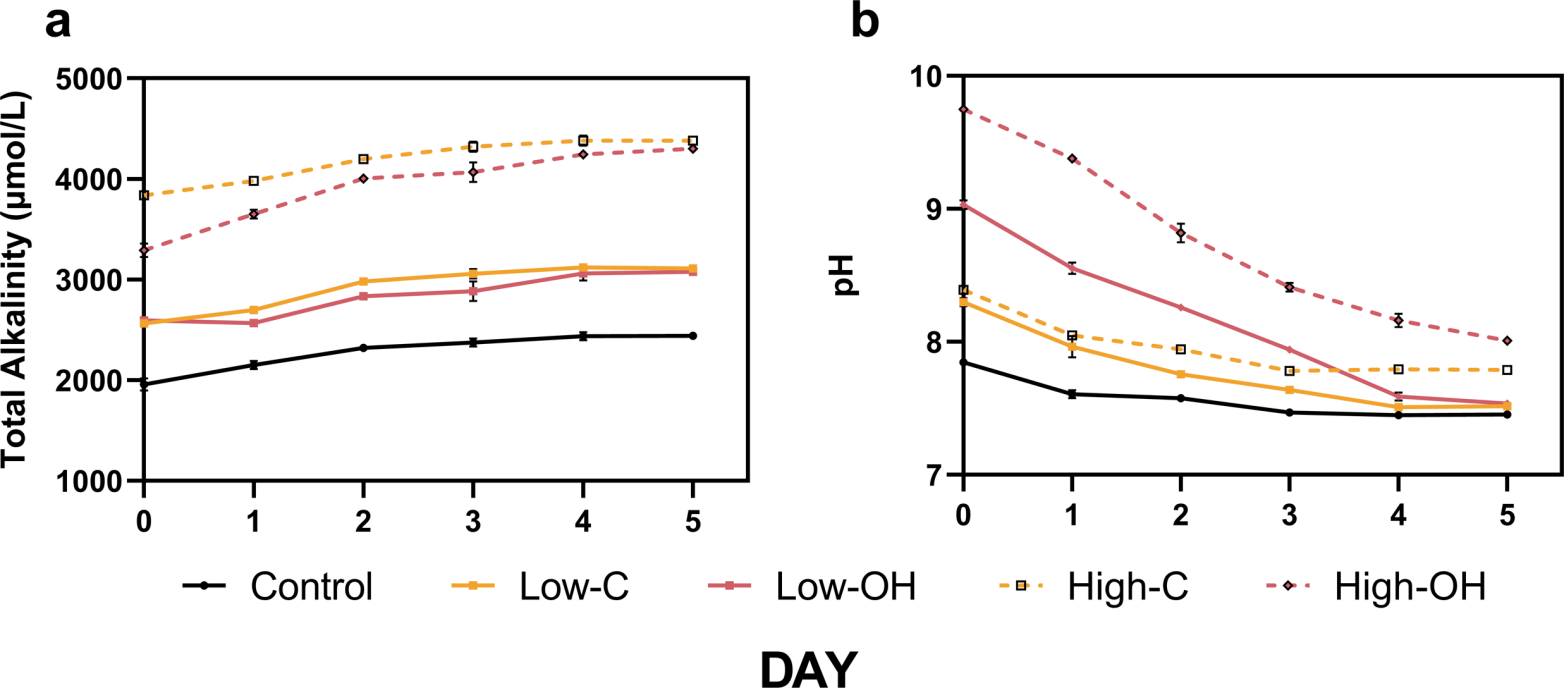
Carbonate chemical parameters in the culturing medium. Changes in total alkalinity (TA, **a**) and pH (**b**) during 5-day culturing of HNFs and heterotrophic bacteria in the 0.2‰ final concentration yeast extract natural seawater culture medium. Day 0 is the transfer day.

On day 0, the TA of the Control, Low-C, Low-OH, and High-C were, respectively, 1,959.0 ± 62.2, 2,563.5 ± 12.1, 2,595 ± 10.0, and 3,838.5 ± 40.0 µmol L^−1^. However, the TA level of high-level NaOH treatment was 3,290.7 ± 69.1, which was lower than expected due to the chemical precipitates that were removed by filtration before the TA measurements (Fig. 2a). From Day 0 to Day 2, pH declined due to CO_2_ absorption from the atmosphere, and cell respiration (Fig. 2b) in the NaOH treatments, resulting in dissolution of precipitates and a sharp rise of the TA compared to other treatments. The TA of treatments with the same TA level were close to each other on Day 2. Then, the TA of all treatments was stable.

Compared to TA, pH showed a larger difference from the beginning to the end (Day 5, Fig. 2b). The initial pH in the Control medium was 7.846 ± 0.027. As a strong base, adding NaOH raised the pH higher than the other treatments: Low-OH and High-OH were 9.032 ± 0.034 and 9.749 ± 0.006, respectively. For NaHCO_3_ addition treatments, pH was lower than NaOH treatments and higher than the Control: Low-C and High-C were, respectively, 8.299 ± 0.029 and 8.391 ± 0.026. During the 5-day culturing period, the pH decreased in all treatments, particularly in the NaOH-added media (Fig. 2b).

In summary, the two substances demonstrate distinct carbonate system dynamics in the culture media. Both NaHCO₃ and NaOH elevated TA, which is crucial for enhancing the ocean’s capacity to sequester CO_2_. However, NaOH also sharply increased pH, which can affect the physiological conditions and transcriptomic activities of HNFs more significantly as an environmental stress.

### HNF responses to OAE under acute exposure

**Fig 3:**
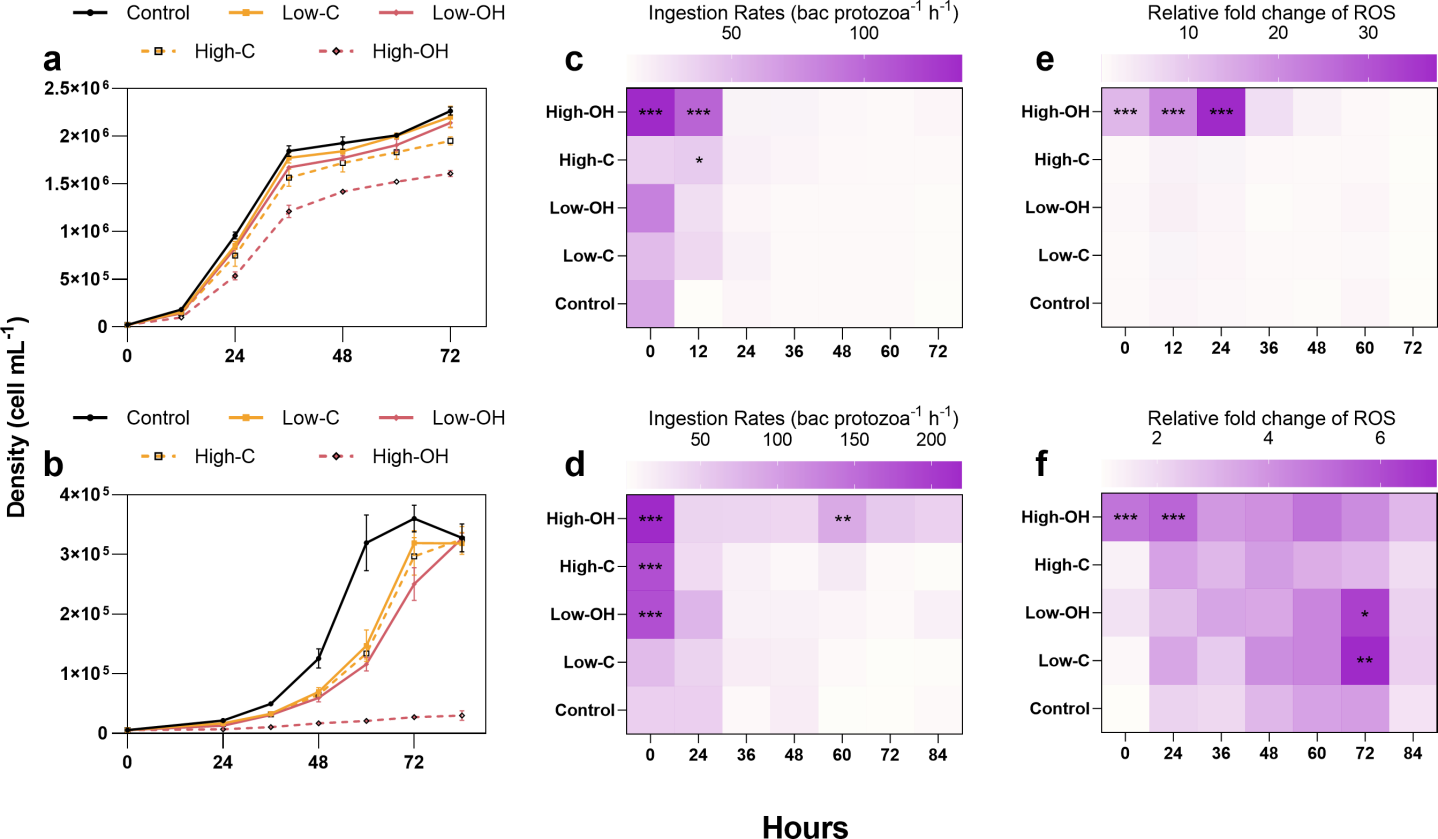
Physiological responses of the HNFs to OAE in the Acute experiment. (**a and b**) Response of CB (**a**) and PL (**b**) density to OAE in the Acute experiment period. The error bar indicates the SD of the mean from the biological triplicates. (**c through f**) Heatmaps showing the response of ingestion rates of CB (**c**), PL (**d**), and the oxidative stress quantified with ROS of CB (**e**), and PL (**f**) to OAE in the Acute experiment period. The ROS level in Control at T0 was set as 100% for each species. Significant differences between the Control and each OAE treatment at each sampling time are given by *P*-value (mixed-effects models; *P < 0.05, **P < 0.01, ***P < 0.001).

All OAE treatments inhibited the growth of HNFs and significantly affected their ingestion rates and ROS production under acute exposure, in which the high-level enhancement and the addition of NaOH showed more negative effects. CB and PL responded similar to acute OAE exposure (mixed-effects models, Fig. 3).

The cell densities of both CB and PL were lower under all OAE treatments, with Low-C, Low-OH, and High-C resulting in similar inhibition effects (Fig. 3a and b). Even though High-OH was the most adverse treatment for both species, CB demonstrated higher tolerance than PL. CB under High-OH showed a sigmoid pattern and reached the plateau at around 48 h, which was the same as the other four treatments (Fig. 3a). PL density in the control got to the plateau stage at 60 h, which was around 12 h earlier than that in Low-C, Low-OH, and High-C. The high level of NaOH addition led to a linear growth pattern of PL (Fig. 3b). Consistent with the cell density change over time, High-OH was also the most significant treatment for ingestion and ROS for the two species. The high-level addition of NaOH increased the ingestion rates of CB (0, 12 h) and PL (0, 60 h) and also induced CB (0, 12, 24 h) and PL (0, 12 h) to accumulate more ROS (Fig. 3 through f).

### HNFs physiological responses to OAE after acclimation

**Fig 4:**
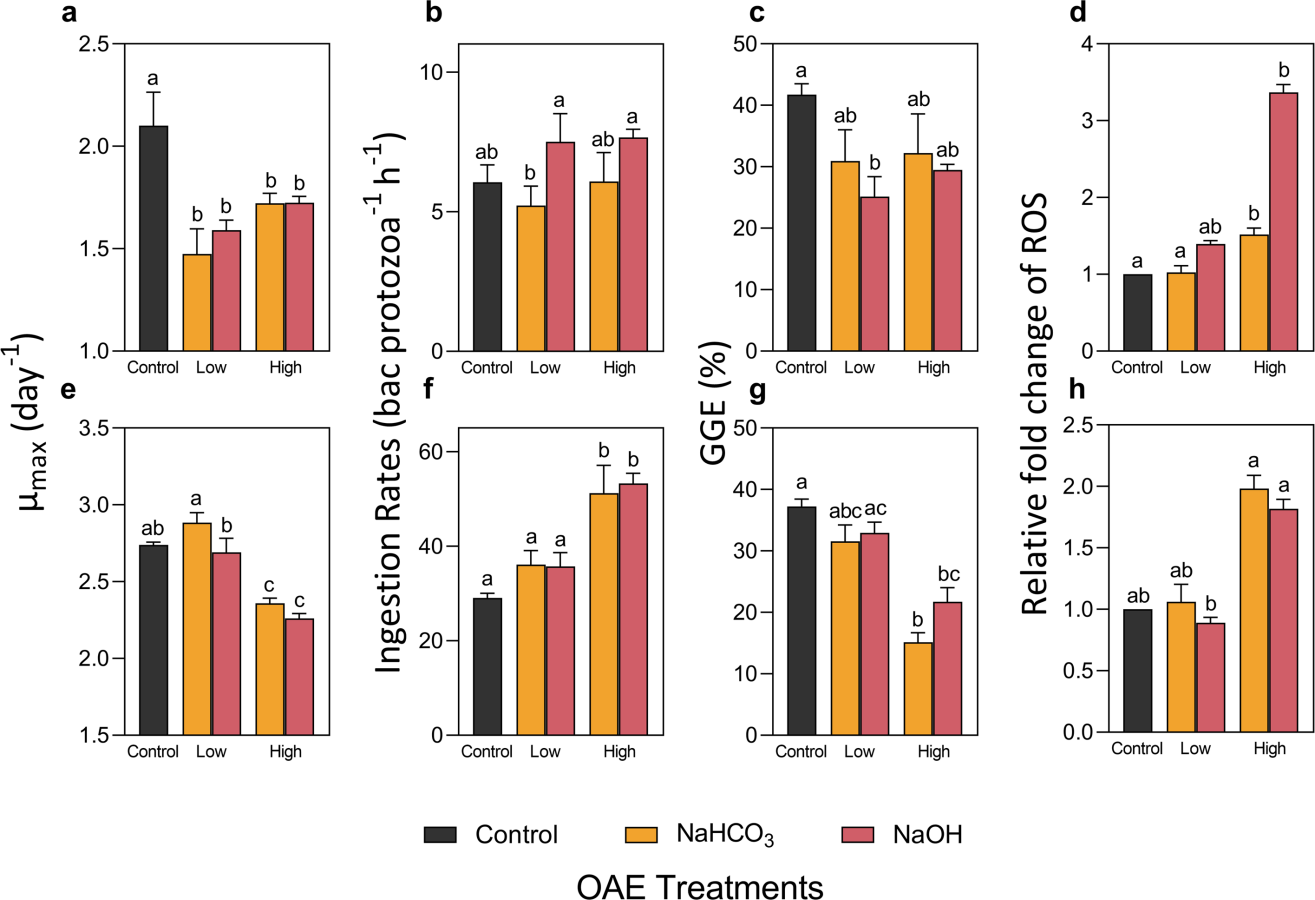
Physiological responses of the HNFs to OAE in the Acclimated experiment. Maximum growth rates (*μ*_max_, **a and e**), ingestion rates when the growth rates were *μ*_max_ (**b and f**), the corresponding gross growth efficiencies (GGE, **c and g**), and the relative fold change of reactive oxygen species at μmax (ROS, **d and h**). The error bar indicates the SD of the mean from the biological triplicates. Physiological values of CB are shown in (**a through d**) and PL in (**e through h**). The alphabet represents the significant differences among treatments, with the same alphabet in multiple comparisons indicating no significant difference between treatments (Tukey’s test and Dunn-Bonferroni test, 95% family-wise confidence level).

After more than 30 generations of acclimation to OAE, we observed significant effects on maximum growth rates, ingestion rates, and GGE in the two species studied, each exhibiting species-specific responses (ANOVA & Kruskal-Wallis test; Tukey’s & Dunn’s tests, Table 1, Fig. 4).

**TABLE 1:** Results of statistical analysis on the effects of OAE in the Acclimated experiment on the physiological traits (i.e., maximum growth rates [*μ*_max_], ingestion rates, GGE, and relative fold changes of ROS) of the two HNF species, by a one-way ANOVA or Kruskal-Wallis test

Parameter	Acclimated OAE		
	Species	df	*P^a^*
*μ*_max_	CB	4	<0.001***
	PL	4	<0.001***
Ingestion rate	CB	4	0.001949**
	PL	4	<0.001***
GGE	CB	4	0.01053*
	PL	4	0.001756**
ROS	CB	4	0.001543**
	PL	4	0.01556*

^a^ Significance: *P < 0.05, **P < 0.01, ***P < 0.001.

Following the acclimation period, the maximum growth rates of CB were markedly hindered by OAE, regardless of the enhancement levels and substances (Tukey’s test, Fig. 4a). The ingestion rates among treatments did not show differences (Tukey’s test, Fig. 4b). When combining the *μ*_max_ and ingestion rates, the GGE was consistently lower in the presence of OAE treatments although only Low-OH showed statistically significant differences (Dunn test, Fig. 4c). CB accumulated more ROS under OAE conditions, with an increase observed under the high-level treatments (Dunn test, Fig. 4d).

PL could acclimate to OAE better than CB, especially in the low-level enhancement treatment. There were no notable variances in *μ*_max_, ingestion rates, GGE, and ROS between the low-level OAE treatments and the Control (Dunn test, Fig. 4e through h). PL could also not acclimate well to the high-level OAE treatments. The high pH and TA levels increased their prey intake, yet they still could not maintain a normal growth rate, and the GGE was inhibited under high-level OAE (Dunn test, Fig. 4e through g). The relative fold change of ROS was increased under high-level OAE, but not significantly (*P* = 0.0585 and 0.1427 for High-C and High-OH, respectively; Dunn test, Fig. 4h). In conclusion, PL could retain the growth efficiency and ROS level under low-level enhancements, but the efficiencies were significantly decreased under high-level OAE.

### Molecular mechanisms underlying physiological responses of low-level acclimated HNFs

**Fig 5:**
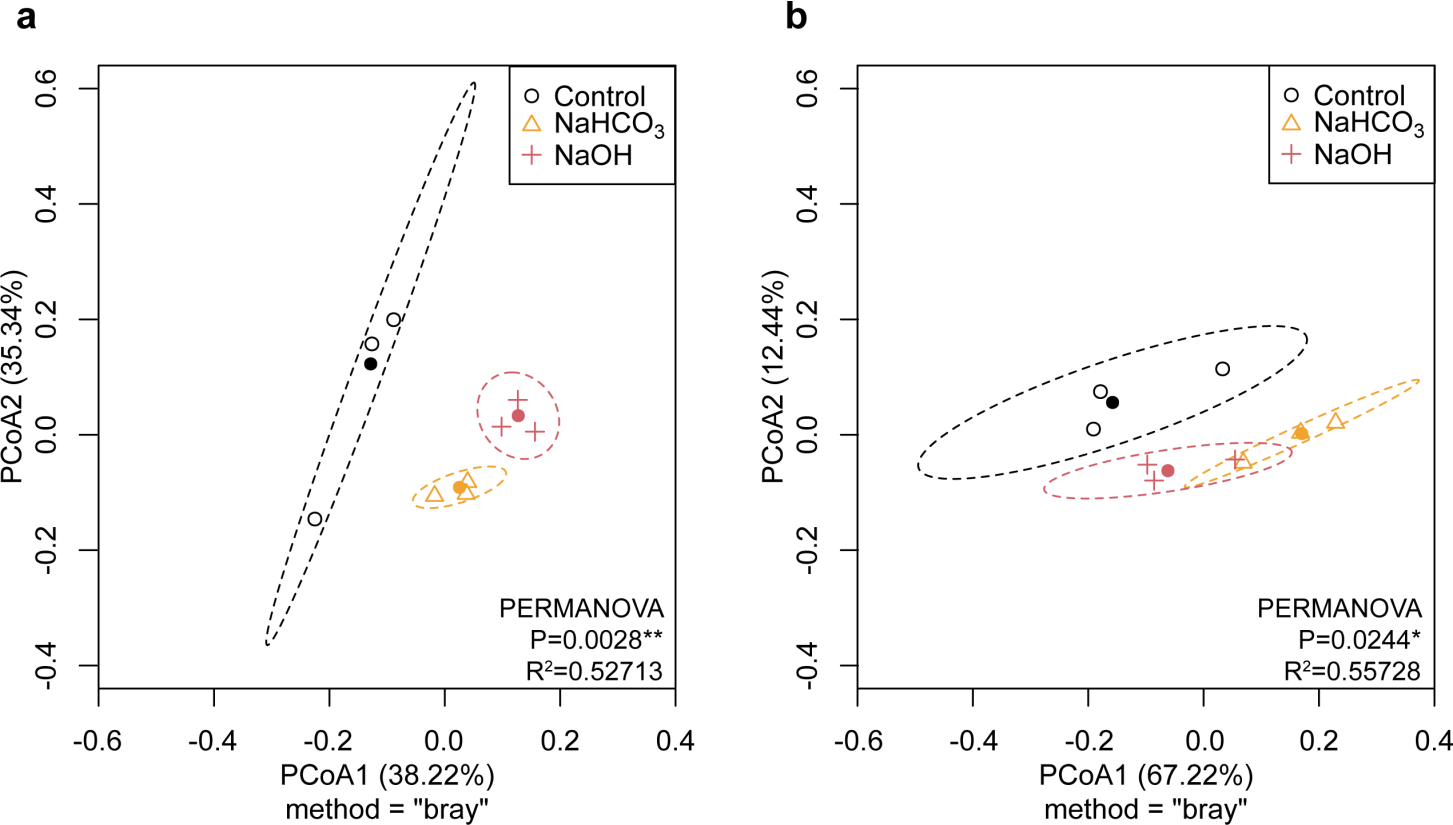
Principal coordinates analysis (PCoA) figures based on the transcriptomic expression patterns of the Control, low-level NaHCO_3_, and low-level NaOH OAE samples of CB (**a**) and PL (**b**). The dashed ellipses represent 95% confidence intervals for the OAE treatment groups.

To investigate if the physiological responses were supported by molecular evidence and how the HNFs were affected by low-level OAE, which is more feasible than the high-level in the real ocean, we compared the gene expression among treatments. For CB, significant differentiation in Control, Low-C, and Low-OH sample transcripts was shown by PCoA plot, where the three experimental groups were clustered separately with significant differences (*P* < 0.05, PERMANOVA, Table S2; Fig. 5a). The PCoA plot indicated that the Control group was distinctly separate, whereas the Low-C and Low-OH samples exhibited some overlap regarding PL (Fig. 5b).

**Fig 6:**
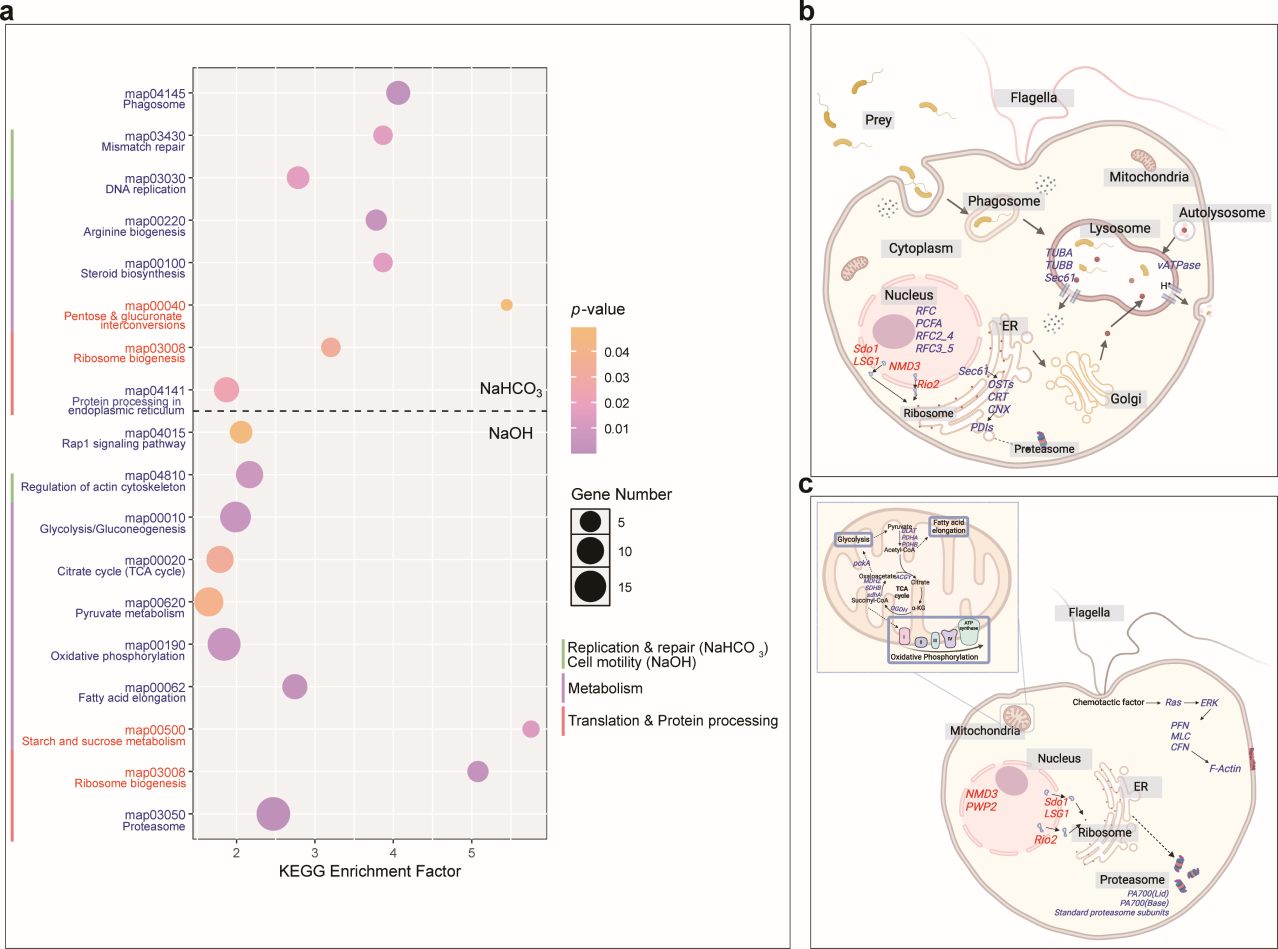
Results of transcriptomic responses of Low-level OAE acclimated CB. (**a**) Bubble plots of CB indicate the *P*-value, KEGG pathway, and enrichment factor of each pathway. KEGG, Kyoto Encyclopedia of genes and genomes. (**b and c**) Schematic representation of CB after the low-level NaHCO_3_ (**b**) and low-level NaOH (**c**) OAE acclimation. Upregulated pathways and genes are highlighted in red, and downregulated pathways and genes are highlighted in blue.

For CB, the BUSCO completeness of the transcriptome assembly was 80.0% [S: 68.0%, D: 12.0%], F: 8.0%, M: 12.0%. Compared to the Control, Low-C resulted in a total of 1,237 DEGs, in which 475 (38.40%) genes were significantly downregulated and 762 (61.60%) genes were upregulated (Table S3). DEGs were significantly enriched (*P* < 0.05) in eight pathways, including six downregulated and two upregulated ones (Fig. 6a and b; Table S3). For Low-OH, a total of 2,367 DEGs were detected, with 966 (40.81%) downregulated and 1,401 (59.19%) upregulated. There are eight pathways significantly downregulated compared to the Control, and only ribosome biogenesis and starch and sucrose metabolism were upregulated (*P* < 0.05; Fig. 6a and c; Table S3). Taking into account the interactions of biological processes across (sub)cellular organelles, we further examined the genes that were significantly up- and downregulated in metabolism (arginine biosynthesis, steroid biosynthesis, pentose and glucuronate interconversions, fatty acid elongation, glycolysis/gluconeogenesis, oxidative phosphorylation, TCA cycle, pyruvate metabolism, starch and sucrose metabolism), replication & repair (DNA replication, mismatch repair) for cell proliferation, translation & protein processing (ribosome biogenesis, protein processing in endoplasmic reticulum, proteasome), and phagosome representing ingestion. Overall, the transcriptomic analysis reveals that CB exhibited a predominant downregulation of critical cellular processes, suggesting an impaired capacity to sustain normal physiological functions and indicating a potential compromise in acclimate resilience under low-level OAE exposure.

**Fig 7:**
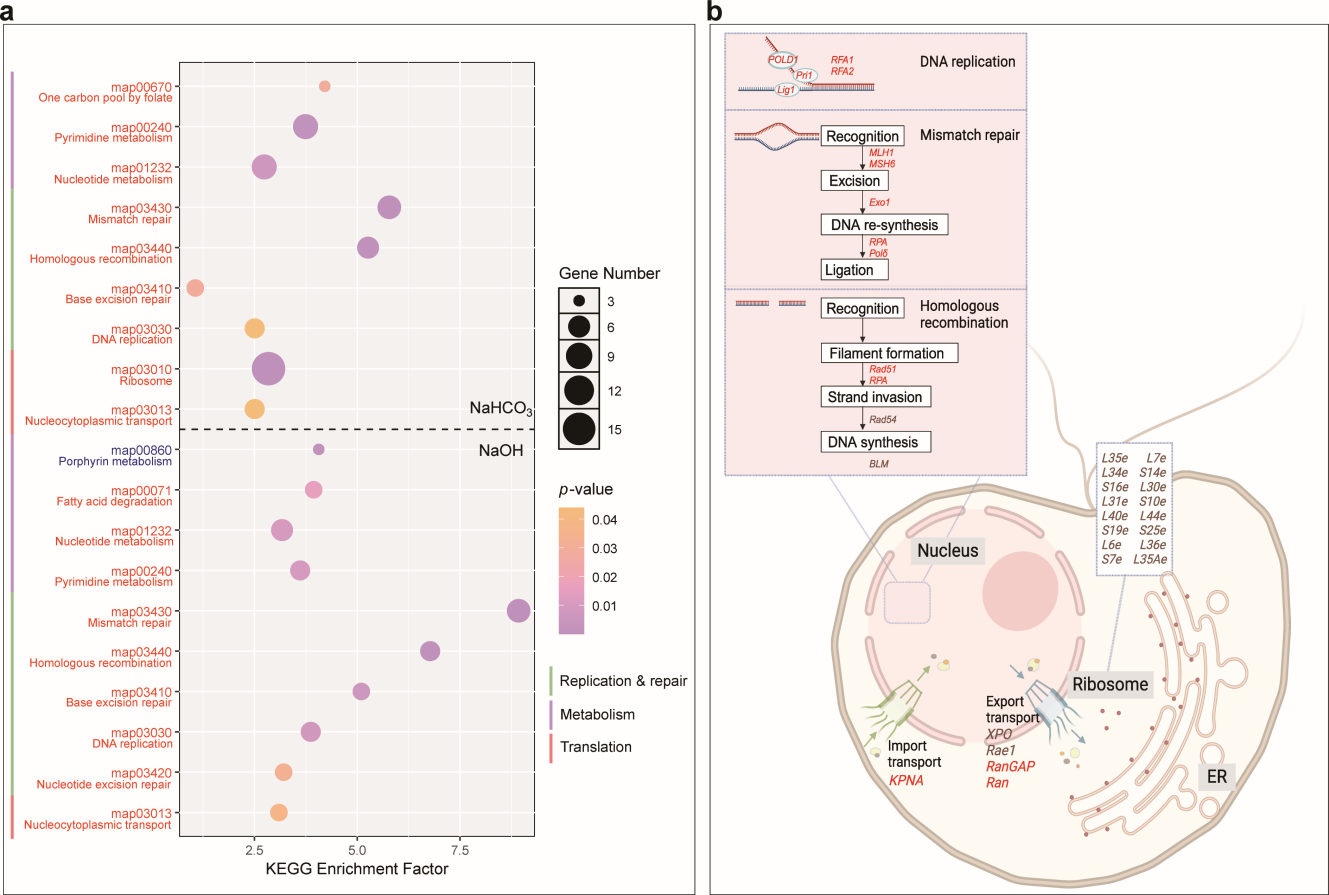
Results of transcriptomic responses of Low-level OAE acclimated PL. (**a**) Bubble plots of PL indicate the *P*-value, KEGG pathway, and enrichment factor of each pathway. KEGG, Kyoto Encyclopedia of genes and genomes. Upregulated pathways are highlighted in blue, and downregulated pathways are highlighted in blue. (**b**) Schematic representation of PL after the low-level NaHCO_3_ and NaOH OAE acclimation. Upregulated genes significant in both NaHCO_3_ and NaOH treatment are highlighted in red, and genes only significant in NaHCO_3_ treatment are highlighted in brown.

In the case of PL, the BUSCO completeness of the transcriptome assembly was 96.0% [S: 53.0%, D: 43.0%], F: 2.0%, M: 2.0%. The expression profiles differed significantly (*P* < 0.05, PERMANOVA, Table S2). Relative to the Control, the Low-C condition resulted in a total of 1,969 DEGs, comprising 1,113 (56.53%) significantly upregulated and 856 (43.47%) genes downregulated. The subsequent enrichment analysis showed that these genes were significantly enriched in nine upregulated KEGG pathways (Table S4), which were further grouped into three interrelated biological processes: replication & repair (mismatch repair, homologous recombination, base excision repair, and DNA replication), metabolism (pyrimidine metabolism, nucleotide metabolism, and one carbon pool by folate), and translation (ribosome, nucleocytoplasmic transport) (*P* < 0.05; Fig. 7a and b). For the Low-OH condition, a total of 986 DEGs were identified, with 477 (48.38%) genes upregulated and 509 (51.62%) genes downregulated, following 1 downregulated pathway and 9 upregulated pathways (Table S4). These pathways were also clustered into three groups: replication & repair (mismatch repair, homologous recombination, base excision repair, DNA replication, nucleotide excision repair), metabolism (porphyrin metabolism, nucleotide metabolism, pyrimidine metabolism, fatty acid degradation), and translation (nucleocytoplasmic transport) (*P* < 0.05; Fig. 7a and b). The upregulated pathways indicated that, despite OAE leading to increased mistakes during replication and chemical pressure, PL responded positively to maintain normal physiological functions.

## DISCUSSION

Understanding the effects of OAE on marine heterotrophic nanoflagellates is crucial for informing potential *in situ* OAE activities, as different species may exhibit varied and potentially negative responses to such interventions in their natural environments. To this end, we conducted both acute and acclimated experiments in the laboratory, examining the impacts of low- and high-level OAE with two substances, NaHCO_3_ and NaOH, on two HNFs’ physiological performances and the underlying transcriptomic mechanisms. Our findings revealed that, under acute exposure, both species exhibited similar patterns. All OAE treatments hindered growth, with high-level NaOH being the most detrimental treatment, which increased ingestion and ROS accumulation. Following acclimation, species PL demonstrated greater tolerance and acclimation capacity to low-level OAE than species CB; however, both species negatively responded to high-level OAE. The transcriptomic analyses supported these physiological observations, showing that significantly regulated pathways were predominantly downregulated in CB and upregulated in PL, highlighting their different adaptive abilities. Furthermore, our results indicated that high-level OAE was more adverse, with NaOH exhibiting more pronounced impacts than NaHCO_3_. To our knowledge, this study is the first report on the effects of OAE on marine HNFs and even protozoa, elucidating the underlying transcriptomic changes involved.

### Responses under acute exposure stress

Many heterotrophic nanoflagellate (HNF) species have demonstrated the ability to thrive in a variety of environments, including the deep sea, brackish waters, and meso- to hypersaline conditions, highlighting their tolerance and acclimation capacities (56, 57). In line with these findings, the effects of low-level OAE on the two species we tested were negligible, regardless of the alkaline substance chosen (Fig. 3). However, the detrimental impacts associated with the high-level treatments may arise from environmental stressors that exceed the threshold at which HNFs can maintain normal physiological functions. Since High-C and High-OH were set at the same TA level, the TA of High-OH was closer to that of the Control due to precipitation (Fig. 2). In addition, Xin et al. (58) demonstrated that the biomass of microzooplankton was unaffected by TA. Thus, the more pronounced effects of NaOH can be ascribed to its elevated pH. Although both CB and PL were able to proliferate, cell densities were significantly lower in the High-OH treatment, where pH exceeded 9.5 (Fig. 3a and d). Our understanding of how elevated pH affects the growth and survival of marine HNFs remains limited, with only a few laboratory studies focusing on other protozoa groups, such as ciliates and heterotrophic dinoflagellates. The varying pH tolerances observed among different protozoan species align with previous findings (29, 59). For instance, dinoflagellate *Gyrodinium dominans* growth limits ranged from 8.8 to 9.3, while *Oxyrrhis marina* could thrive at pH above 9.5. Thus, results obtained from our acute experiments are consistent with and reflect the established knowledge regarding the pH tolerance of protozoa. The low densities observed in the High-OH treatment may be attributed to either a species-specific response to high pH or the synergistic stress resulting from the combined effects of elevated pH and TA. We conducted measurements of the respiration rates only at 24 and 48 h for CB and PL, respectively, due to the limitations of the equipment (Fig. S4a and S4b). The metabolic activity (in terms of respiration) was inhibited significantly under the High-OH treatment for both species, indicating the potential disruption of metabolic functions. Thus, HNFs could not obtain enough energy for cell growth through respiration.

In response to this stress, CB and PL showed significantly higher ingestion rates as a compensation mechanism to gather more resources under the unfavorable conditions (Fig. 3b and e), as previously described for scarce nutrients through accelerated food uptake (60). Interestingly, the lower density of PL under High-OH resulted in a higher bacteria: HNFs density ratio in the late phase (after 36 h), suggesting that prey availability was the most adequate in this treatment (Fig. S2). This could explain the significantly higher ingestion rate observed in High-Oh at 60 h, as HNFs had access to a more favorable prey density besides the stress conditions. However, this increased ingestion did not translate into growth, likely due to the detrimental effects of ROS accumulation, which can result from oxidative stress under high pH conditions (Fig. 3c and f). ROS, such as superoxide and peroxides, can form from O_2_ during oxidative phosphorylation in the respiration process, which will accumulate under stress and lead to oxidative stress (61). The significant rise in ROS levels indicates that the cells are experiencing oxidative damage, which can hinder vital cellular functions and contribute to growth inhibition (62). The observed decrease in respiration rates (Fig. S4b) suggests cells may be entering a state of reduced metabolic activity as a protective response to mitigate damage from ROS. Furthermore, the accumulation of ROS is physiologically significant for HNFs as it serves as an indicator of cellular stress that can disrupt essential cellular processes, including energy production and metabolic regulation. Elevated ROS levels can lead to oxidative damage of protein, lipids, and DNA, ultimately impairing growth and reproductive success, which may compromise the overall fitness and ecological role of these organisms in marine ecosystems (63). Collectively, these physiological responses illustrate how high pH conditions can disrupt cellular homeostasis, leading to inhibited growth while triggering compensatory behaviors that ultimately fail to sustain cell viability under oxidative stress. The potential mechanisms underlying these effects will be discussed in the following transcriptomics section.

### Acclimation of HNFs and the underlying mechanisms

Mechanisms underlying physiological performance also proved that PL faced low-level OAE more positively than CB (Fig. 5 through 7). Ribosome biogenesis was the only common upregulated pathway for both Low-C and Low-OH CB samples, which may reflect an attempt to enhance protein synthesis in response to environmental stress (64). However, while elevated ribosome levels may facilitate increased protein synthesis, they could also lead to the accumulation of misfolded proteins or inefficient cellular processes. The downregulation of protein processing in the endoplasmic reticulum under Low-C suggested that newly synthesized proteins may not be properly folded or modified. Moreover, proteasomes, vital in regulating proteins that control cell-cycle progression and apoptosis (65), were downregulated in Low-OH, impairing the degradation of damaged or misfolded proteins. These transcriptomic changes may negatively impact cell proliferation.

Lower ingestion rates with NaHCO_3_ addition can be attributed to the downregulation of phagolysosome-related pathways. Notably, the V-type H^+^-transporting ATPase subunit A (encoded by the gene *vATPase*), which regulates cytosolic and lysosome pH, was downregulated in both treatments (66). This indicates the challenges posed by high pH and TA in maintaining cellular pH and homeostasis (Table S3 and S4). This finding aligns with a previous study demonstrating that dinoflagellates *Amphidinium carterae* and *Hetrocapsa oceanica* struggled to control intracellular pH and disrupt growth under environmental pH changes (67).

Under the Low-OH condition, 10 DEGs involved in the TCA cycle and 17 DEGs involved in oxidative phosphorylation were significantly downexpressed. These pathways are essential for ATP production (68), and their downregulation may disrupt the balance between ROS production and removal (34). Specifically, the downregulation of multiple genes involved in oxidative phosphorylation, including subunits of Complexes I (*NDUFA12*, *NDUFAB1*, *NDUFS7*, *NDUFV2*), II (*SDHB*), III (*CYC1*, *CYTB*), and IV (*COX17*), as well as ATP synthesis components (Fig. S5). This pattern of gene downregulation suggests significant mitochondrial dysfunction, leading to impaired electron transport and increased ROS production through mechanisms such as electron backup at Complex I and III, as well as potential reverse electron transfer from succinate accumulation (34, 69, 70). Consequently, these findings highlight the complex interplay between mitochondrial dysfunction and oxidative stress in response to Low-OH conditions. In response to environmental stressors like OAE, cells may produce increased ROS as byproducts of altered metabolic processes. While elevated ROS levels can act as signaling molecules that trigger further stress responses, excessive ROS can lead to oxidative damage (35), potentially hindering CB’s competitive ability in future NaOH-based OAE environments.

In contrast to CB, PL upregulated pathways associated with various cellular processes, despite no significant physiological changes being witnessed (Fig. 4), with only marginal differences observed between NaOH and NaHCO_3_ (Fig. 5 and 7). Both Low-C and Low-OH samples exhibited five DEGs significantly upexpressed in DNA replication, which could enhance cell division and improve replication accuracy (71). However, the growth rates we measured were comparable to Control, indicating a trade-off between proliferation and maintenance under stress. The ingestion rate in Low-C was higher than that of the control. The observed changes in lysosomal pathway gene expression indicate a complex relationship with ingestion rates (Fig. S5), with some genes upregulated (*AP3B*, *CTSD*) and some downregulated (*DNASE2*, *SCARB2*, *SUMF1*). Thus, the net effect on ingestion rates mostly depends on the upregulation of endocytosis genes (*CHMP1*, *PDCD6IP*, *VPS28*, *VTA1*). Additionally, repair-related pathways were significantly upregulated (Fig. 7), which are crucial for the detection and repair of DNA errors and damage. The upregulation of DNA repair pathways suggests that PL is actively prioritizing the maintenance of genomic integrity in stress response, which is essential for sustaining normal physiological functions and preventing growth inhibition (72, 73). Similarly, the enhancement of nucleocytoplasmic transport facilitates the efficient movement of proteins and RNA, allowing for timely responses to environmental changes and ensuring that essential cellular processes continue to function optimally (74, 75). Together, these acclimations indicate that PL may be prioritizing DNA repair and replication in response to stress rather than focusing on growth, leading to a focus on maintenance over proliferation. Furthermore, nucleocytoplasmic transport, another upregulated pathway in the two low-level treatments, was also a positive response that facilitated the movement of proteins and RNA, thereby increasing the efficiency of gene expression in response to environmental changes (76). Ribosome activity was also increased in the Low-C condition, allowing the production of more proteins for essential cellular functions and OAE acclimation.

### Technological and ecological meaning of HNF acclimation to OAE

This study focused on the effects of two chemicals, highlighting the basic and common impacts on marine organisms while intentionally excluding trace metals and nutrients. The primary distinction between the two substances was the pH levels. While the addition of sodium bicarbonate primarily elevated TA without significantly altering pH, the sodium hydroxide treatments resulted in substantial increases in both parameters. This dual enhancement can create a more stressful environment for heterotrophic nanoflagellates, as elevated pH can exacerbate the physiological impacts of increased TA. These findings underscore the importance of carefully selecting alkaline materials to optimize conditions for marine life. Naturally occurring materials from different source regions may contain various elements (3) and can have varying effects on protozoa and marine ecosystems. For example, when limestone, olivine, or magnesite are used for *in situ* input, the main elements released are Mg and Ca ions, along with trace metals like Fe, which may be sufficient to induce toxicity (14, 77). Hence, OAE may become a novel environmental anthropogenic pollution, and consideration of the potential impacts of different alkalines is necessary for the next step study. Moreover, the high-level enhancements were significantly more harmful than the low-level conditions. In fact, the low-level enhancement is more likely to be implemented in the ocean, while the high-level enhancement is only an extreme laboratory condition. However, we sound an alarm that the addition levels in the natural ocean should be prudently evaluated before implementing OAE. Besides, determining the minimum inhibitory concentrations (MICs) for various OAE substances is crucial, as understanding these thresholds will guide the safe application of OAE and help mitigate potential adverse effects on marine organisms, thereby providing essential data to inform stakeholders and policymakers in their decision-making processes regarding the real-ocean OAE implementation.

Several ecological implications emerge considering the observed species-specific acclimation to OAE. As OAE continues to progress, it is imperative to explore the cumulative effects of chronic exposure to varying TA, pH levels, and chemical additives on protozoan physiology and ecology. Fragmentation of habitable areas may occur due to certain geological and climatic conditions, potentially favoring organisms with specific adaptations (78). In this context, organisms that are able to acclimate to OAE conditions, like PL, may become more dominant than ones like CB. Furthermore, the biological responses of protozoa to environmental changes can serve as indicators for the response of higher organisms, as protozoa share characteristics with eukaryotic cells (79). Based on our results, the deleterious effects of OAE may extend to higher creatures.

In addition, through their ability to move nutrients and energy from primary producers and bacteria from the microbial loop to higher trophic levels, HNFs are essential to the functioning of the marine food chain (80, 81). Changes in the abundance and activity of protozoa due to OAE may influence the dynamics of the entire food web. Based on our results, PL, which demonstrated a competitive advantage under low-level OAE treatments, has a diameter about twice that of CB. This suggests that OAE could potentially shift the size distribution within HNF communities toward larger taxa, assuming other small HNFs do not acclimate positively. In marine planktonic food webs, where grazing is strongly governed by size-selective predation with predator:prey size ratios spanning orders of magnitude (82), such a shift could alter energy transfer pathways. Specifically, a community dominated by larger HNFs might favor different suites of micro- and meso-zooplankton grazers, depending on their prey size preferences and ability to switch prey. The net effect would, thus, depend on the acclimate ability of both the remaining HNF prey and their predators.

Moreover, the unfavorable reactions of HNFs to OAE may have broader implications for the global carbon cycle, as they are major contributors to the remineralization of organic matter in the ocean (83, 84). This potential slowdown in remineralization could disrupt the efficiency of the biological carbon pump, which relies on the continuous cycling of nutrients to support phytoplankton growth and, in turn, carbon sequestration in the deeper ocean (85). Our work provides foundational data on the impact of OAE on HNFs, highlighting the need for further investigation into other protozoa, related organisms, and the overall marine ecosystem. Future studies should also consider the interactive effects of different environmental stressors, such as temperature, to provide a more comprehensive understanding of how OAE may shape marine ecosystems.

### Conclusion

Our study provides crucial insights into the effects of ocean alkalinity enhancement on marine heterotrophic nanoflagellates, revealing substantial physiological and transcriptomic responses to varying levels of NaHCO_3_ and NaOH. The differential tolerance shown by *Cafeteria burkhardae* and *Paraphysomonas longispina* underscores the complexity of species-specific responses to OAE. Specifically, after acclimation, *C. burkhardae* exhibited a significant decrease in growth rates across all OAE treatments, with a notable accumulation of ROS at high levels of NaOH. In contrast, *P. longispina* maintained normal physiological performance under low-level OAE treatments, while high-level NaOH exposure still resulted in significantly slower growth and reduced GGE. Heterotrophic nanoflagellates play a vital role in marine ecosystems by facilitating nutrient cycling and energy transfer within the food web. Our results indicate that OAE can significantly harm HNFs, potentially disrupting the dynamics of marine food webs and impacting higher trophic levels. Given the ecological importance of protists, the adverse effects of OAE raise serious concerns regarding marine biodiversity and ecosystem stability, and our findings emphasize the need for careful consideration of in-situ OAE implementation. While these results provide valuable insights, caution is practiced when extrapolating our findings to natural systems, as the experiments were conducted in controlled laboratory settings with limited volumes. Additionally, the absence of genomic data for high alkalinity treatments or acute conditions suggests that further research is needed to fully understand the ecological implications of OAE. As climate change mitigation strategies evolve, it is crucial to recognize the ecotoxicology of OAE practices to prevent unintended consequences for marine ecosystems and ensure the health of ocean carbon cycling.

## Data Availability

The RNA-seq data of *C. burkhardae* and *P. longispina* cultures have been deposited in the NCBI database under BioProject numbers PRJNA1192676 and PRJNA1192746, respectively. The authors declare that all supporting data of this study are available within the article and its supplemental material or from the corresponding authors upon request.

## SUPPLEMENTAL MATERIAL

Supplemental figures aem.00298-26-s0001.docx Fig. S1 to S5.

Supplemental tables aem.00298-26-s0002.xlsx Tables S1 to S4.

ASM does not own the copyrights to Supplemental Material that may be linked to, or accessed through, an article. The authors have granted ASM a non-exclusive, world-wide license to publish the Supplemental Material files. Please contact the corresponding author directly for reuse.
