# Supplementary material for: Assessing the effects of ocean alkalinity enhancement on marine protozoa: physiological dynamics and transcriptomic responses
Source: Appl Environ Microbiol. 2026 Jun 30;92(7):e00298-26. doi: 10.1128/aem.00298-26 (PMC13390489; doi:10.1128/aem.00298-26)
Supplement: Supplemental figures — Fig. S1 to S5. [file aem.00298-26-s0001.docx]

Figures:

**Figure S1** Changes in Dissolved Inorganic Carbon (DIC, **a**), *p*CO_2_(**b**), HCO_3_^-^ concentration (**c**), and CO_3_^2-^ concentration (**d**) during the 5-day culturing of HNFs and heterotrophic bacteria in the 0.2‰ final concentration yeast extract natural seawater culture medium. Day 0 is the transfer day.

**Figure S2** Bacterial prey conditions during the Acute experiment in CB (**a** and **b**) and PL (**c** and **d**) culture media. (**a**, **c**) bacteria densities; (**b**, **d**) total bacteria: HNFs ratio during the experiment.

**Figure S3** Growth curves of CB (**a**) and PL (**b**) in the Acclimated experiment period.

**Figure S4** Respiration rates of HNFs to OAE in the Acute (**a** and **b**) and Acclimated (**c** and **d**) experiments.

**Figure S5** Heatmaps of significantly up- and down-regulated genes in ROS and ingestion-related pathways within the OAE treatments compared to the control. (**a**) down-regulated genes of CB; (**b**) up-regulated genes of CB; (**c**) down-regulated genes of PL; (**d**) up-regulated genes of PL.

Tables (in excel):

**Table 1.** Experimental design details.

**Table 2.** Results of statistical analysis on the effects of OAE in the Acclimated experiment on the transcriptomic expression of CB and PL, by PERMANOVA.

**Table 3.** KEGG enrichment analysis on differentially expressed genes for *Cafeteria burkhardae*. KEGG, Kyoto Encyclopedia of Genes and Genomes.

**Table 4.** KEGG enrichment analysis on differentially expressed genes for *Paraphysomonas longispina*. KEGG, Kyoto Encyclopedia of Genes and Genomes.


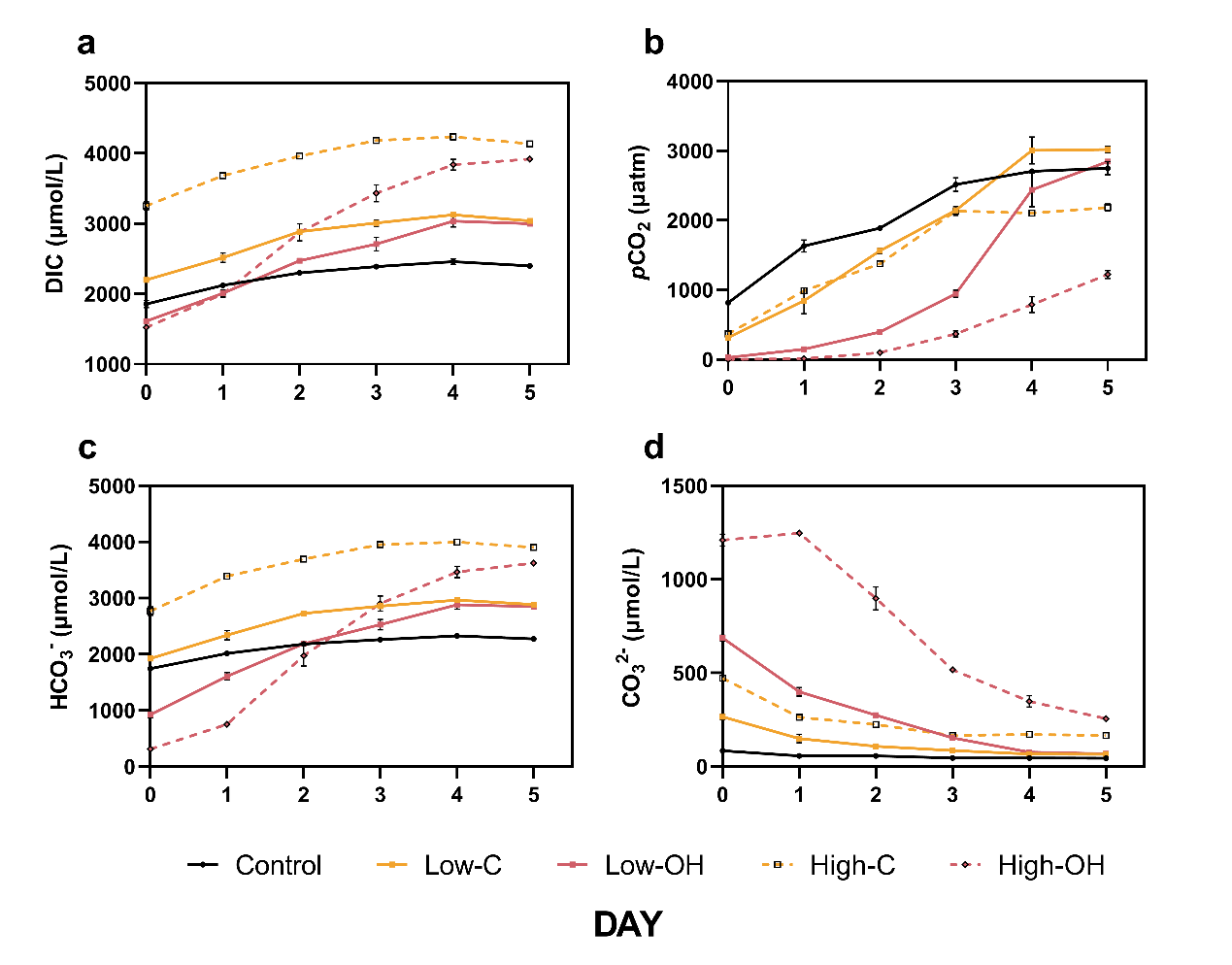


**Figure S1** Changes in Dissolved Inorganic Carbon (DIC, **a**), *p*CO_2_(**b**), HCO_3_^-^ concentration (**c**), and CO_3_^2-^ concentration (**d**) during the 5-day culturing of HNFs and heterotrophic bacteria in the 0.2‰ final concentration yeast extract natural seawater culture medium. Day 0 is the transfer day.


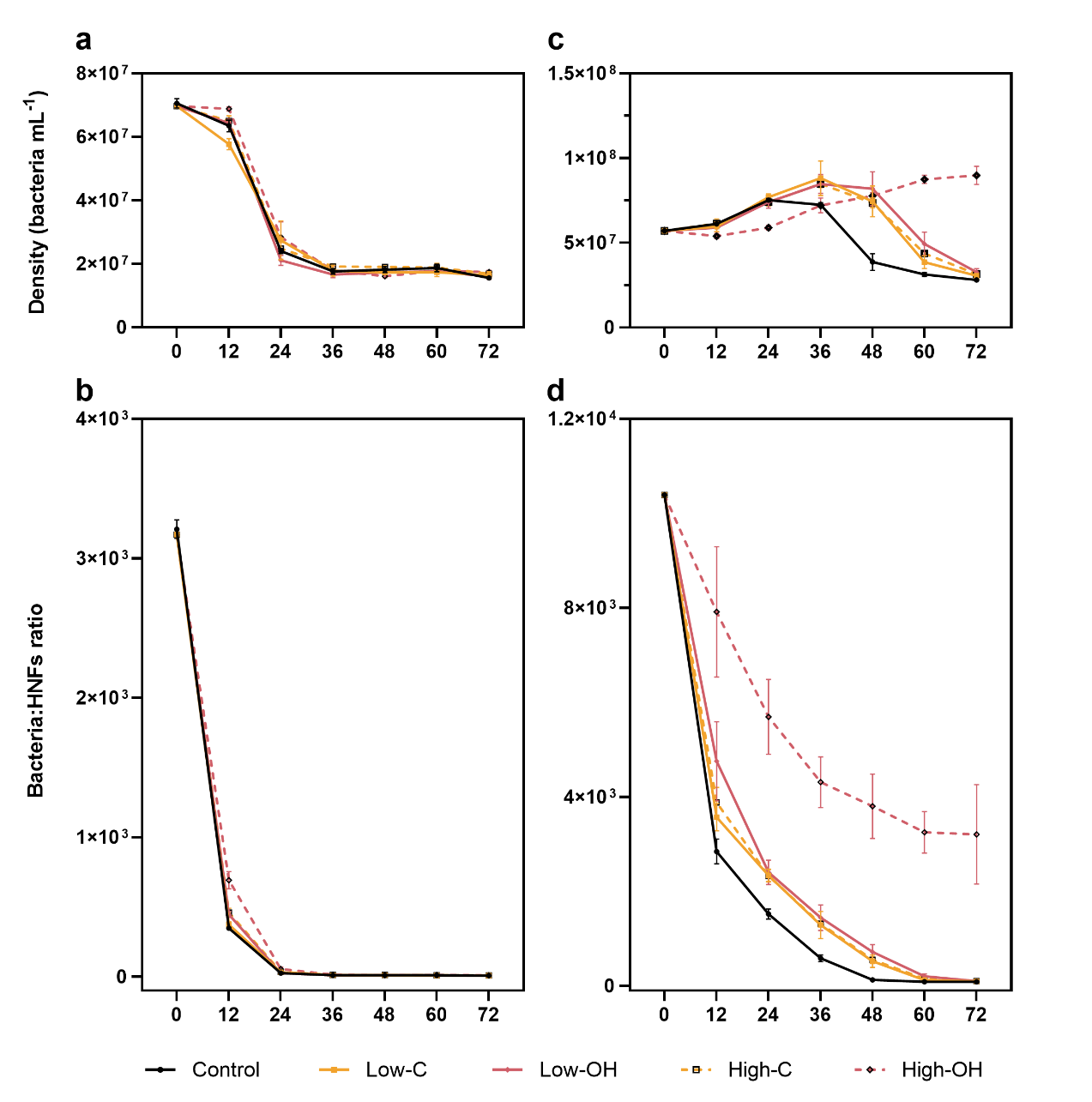


**Figure S2**. Bacterial prey conditions during the Acute experiment in CB (**a** and **b**) and PL (**c** and **d**) culture media. (**a**, **c**) bacteria densities; (**b**, **d**) total bacteria: HNFs ratio during the experiment.


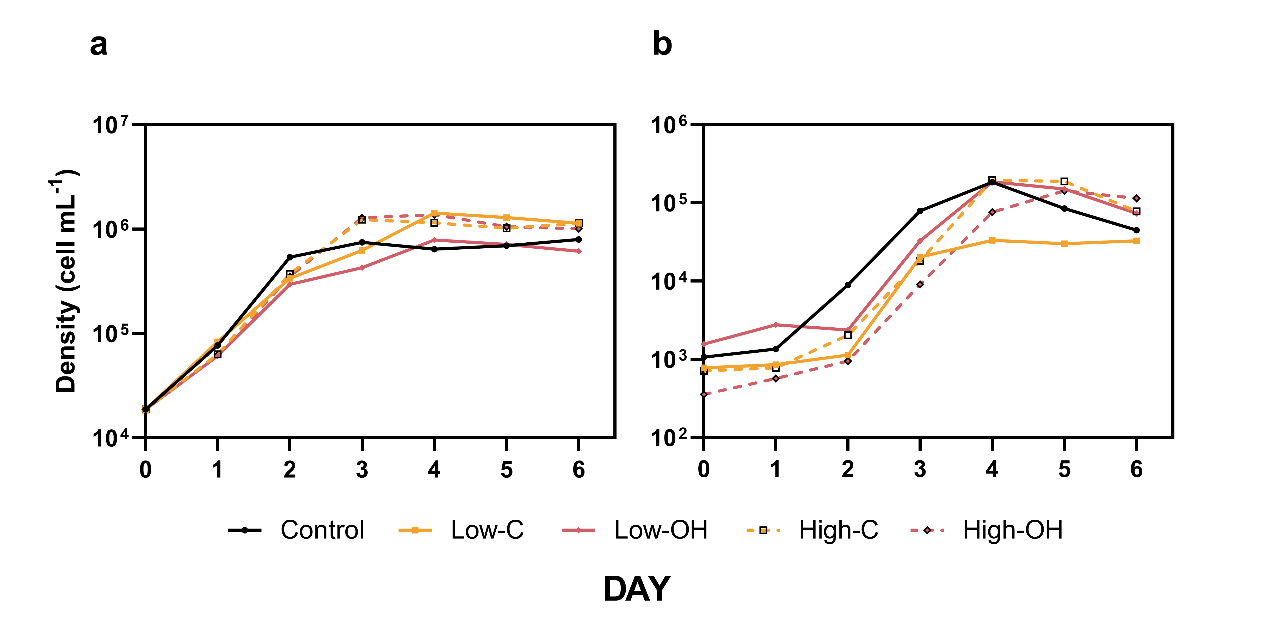


**Figure S3** Growth curves of CB (**a**) and PL (**b**) in the Acclimated experiment period.


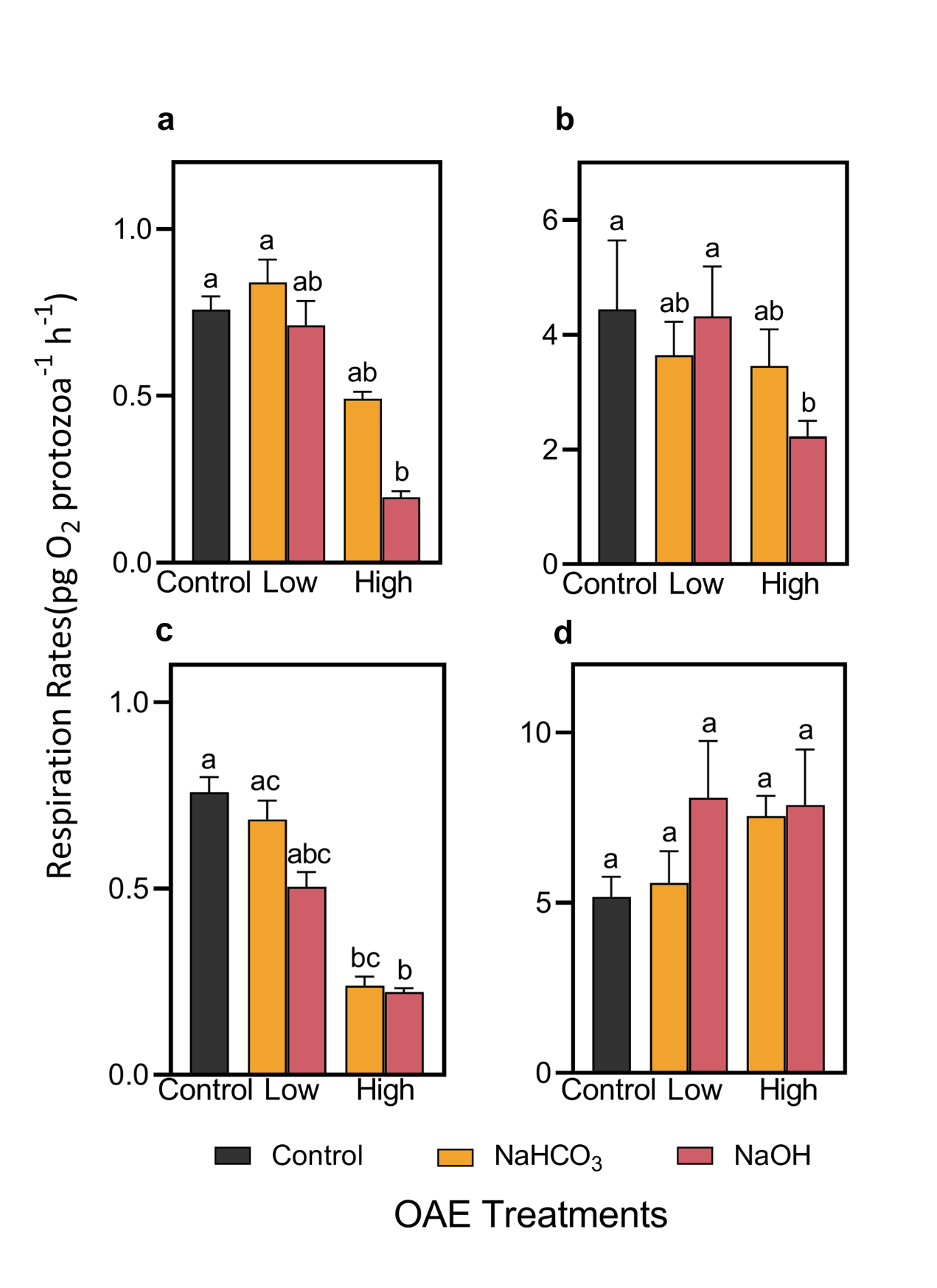


**Figure S4** Respiration rates of HNFs to OAE in the Acute (**a** and **b**) and Acclimated (**c** and **d**) experiments. In which the values of CB are shown in (**a** and **c**) and PL in (**b** and **d**). The error bar indicates the SD of the mean from the biological triplicates. The alphabet represents the significant differences among treatments, with the same alphabet in multiple comparisons indicating no significant difference between treatments (Tukey’s test and Dunn-Bonferroni test, 95% family-wise confidence level). The respiration experiments were conducted in a non-invasive 24-channel oxygen respirometer (SDR-494, SensorDish Reader, PreSens, Regensburg, Germany) at 9 p.m. every day. Two-milliliter cultures in each flask were sucked out and directly transferred into 2 mL glass sensor vials (SV-PSt5-2 mL, PSt5-1749, PreSens, Regensburg, Germany) for the total respiration rates measurement of the culture system including protozoa and bacteria. The control groups were set up by sucking out another 2-milliliter sample and filtering with 1 μm pore size polycarbonate filters (Cytiva™, Whatman) into the same size vials. Then, all the vials were placed on the respirometer to monitor the declines of DO concentration for 3 h at 23 °C in darkness. DO values were measured every 15 seconds during the experiments, and the respiration rates of protozoa were obtained from the differences in oxygen consumption between treatments (protozoa and bacteria) and controls (bacteria only). Samples of the protozoa abundance were taken after the respiration measurements and counted using the same methods as in the growth experiments. The whole cell respiration rates (pg O_2_ protozoa^-1^ h^-1^) of the protozoa were calculated as:

$$\begin{aligned} RR=\frac{\left( DO_{t1}-DO_{t2} \right)-\left( DO_{bt1}-DO_{bt2} \right)}{N_{pro}\times\left( t_{2}-t_{1} \right)}\times{10}^{6}\# \end{aligned}$$

where DO_t1_ and DO_t2_ (μg O_2_ mL^-1^) were respectively the DO level in each treatment (protozoa with bacteria) at the first sampling point t_1_ and the second sampling point t2. DO_bt1_ and DO_bt2_ (μg O_2_ mL^-1^) were the DO levels of the corresponding control (bacteria only) at t_1_ and t2, respectively. N_pro_ (protozoa mL^-1^) was the abundance of protozoa in each treatment, and t_2_ – t_1_ (h) was the sampling interval.


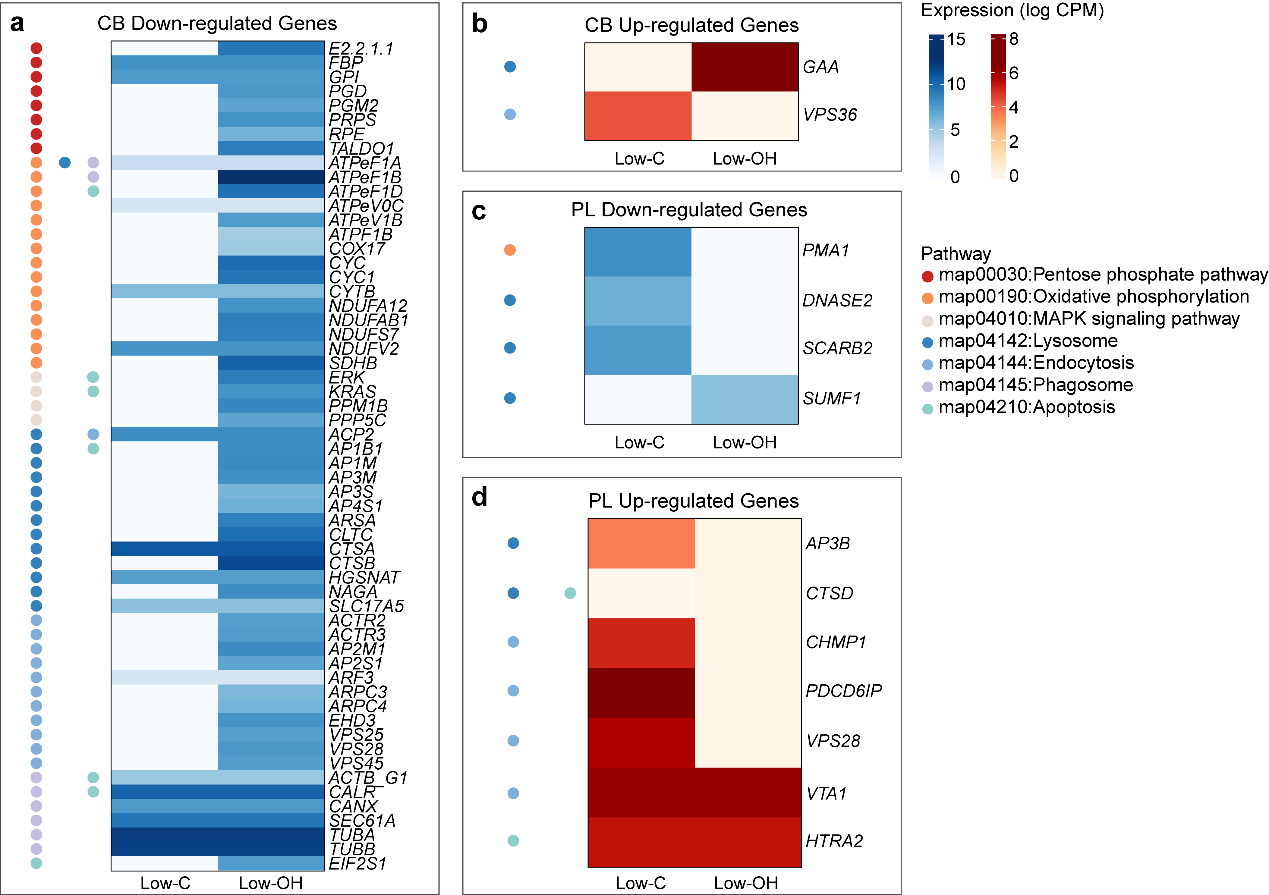


**Figure S5** Heatmaps of significantly up- and down-regulated genes in ROS and ingestion-related pathways within the OAE treatments compared to the control. (**a**) down-regulated genes of CB; (**b**) up-regulated genes of CB; (**c**) down-regulated genes of PL; (**d**) up-regulated genes of PL.
